# Fused Filament Deposition of PLA: The Role of Interlayer Adhesion in the Mechanical Performances

**DOI:** 10.3390/polym13030399

**Published:** 2021-01-27

**Authors:** Sara Liparoti, Daniele Sofia, Aldo Romano, Francesco Marra, Roberto Pantani

**Affiliations:** Department of Industrial Engineering, University of Salerno, Via Giovanni Paolo II, 132, 84084 Fiscian, SA, Italy; sliparoti@unisa.it (S.L.); dsofia@unisa.it (D.S.); aldromano@unisa.it (A.R.); rpantani@unisa.it (R.P.)

**Keywords:** additive manufacturing, polymer crystallization, solidification modeling, molecular diffusion

## Abstract

A set of criteria to enhance mechanical performances of standard specimens (Type V, ANSI D368) made of polylactic acid (PLA) were proposed. Fused PLA deposition was conducted with nozzle temperature ranging from 180 to 230 °C and deposition plate temperature ranging from 70 to 110 °C. Optical microscopy, elastic modulus analysis and density measurement allowed emphasizing the effect of temperature field, also measured during the process, on the morphology and the mechanical characteristics of the specimen. Atomic force microscopy revealed a morphology typical of amorphous samples with globular structures. Poor interlayer adhesion was detected in the part of the specimen located at larger distance from the deposition plate, showing an elastic modulus lower than those measured in the central part (220 MPa vs. 500 MPa). The specimen crystallinity degree was below 3%. The molecular weight between entanglements was adopted as a measure of the interlayer molecular diffusion. A successful diffusion and re-entanglement of the polymer melt at the interface was the key to improving mechanical performance. A mathematical model describing the transient heat transfer during the fused PLA deposition and accounting for solidification and the nonisothermal crystallization kinetics was introduced. Simulated temperature evolutions were consistent with the experimental ones. They were related to the mechanical performances, the morphology, and the molecular weight between entanglements of the parts.

## 1. Introduction

Additive manufacturing techniques have triggered industrial innovations thanks to their ability to realize objects with very complex shapes that are impossible to obtain adopting conventional processes. The additive manufacturing techniques are based on the bottom-up approach, during which the construction of parts is conducted layer by layer. Among the additive manufacturing techniques, fused filament deposition (FDM) is devoted to the production of plastic parts. FDM can be spread out into four main stages: polymer melting inside the extruder, the extrusion through the nozzle, the deposition of the filament on the deposition plate and, subsequently, the construction layer by layer.

Up to now, FDM has been applied to the production of prototypes, medical devices and final parts for automotive and aerospace areas [1,2]. FDM produced parts generally exhibit poor and anisotropic mechanical performances, which make the parts not suitable for industrial applications and where structural properties are required [3]. This drawback has been generally attributed to the layer-by-layer deposition approach. The filament is deposited on a previously deposited layer that is undergoing cooling: this induces the formation of a weld part between adjacent layers. The overall mechanical part resistance depends on the interlayer bond strength [4]. In turn, the interlayer bond strength depends on the molecular diffusion at the interface; in other words, on the possibility of the molecules to recover their entangled organization, also at the interface, after deposition [5]. Such mobility is regulated by the temperature field and the crystallization kinetics. The cooling rate also influences the part properties: if the cooling is too fast, the premature solidification of the polymer hinders molecular interface mobility, and the molecules cannot recover the entangled organization. Furthermore, the crystallization occurring during deposition of a semi-crystalline polymer may freeze the molecules, hindering any molecular diffusion at the interface. It must be pointed out that the crystallization kinetic depends on the flow field experienced during deposition: the stronger the flow, the faster the kinetic [6].

To gain insight into the phenomena taking place during deposition, different approaches have been proposed in the literature. The most common approach is the optimization of process parameters in order to maximize the mechanical performances of the produced parts [7]. However, the wide range of parameters that can be varied makes this approach not suitable for understanding the involved phenomena. Previous research focused on the thermal history of the deposited layers during FDM production since it influences the mechanical properties of the produced parts and it impacts the inter-layers adhesion. Compton et al. [8] recorded thermal evolution of a single-layered structure made of acrylonitrile butadiene styrene during deposition. It was observed that the cooling of a newly deposited layer was very fast, of the order of tens of degrees per second, due to the heat exchange with the environment. When a new layer is deposited on a layer previously deposited, this last undergoes a temperature increase, and then a partial re-melting, due to the heat transfer at the interface [9]. Spoerk et al. [10] correlated the deposition plate temperature to the interlayer adhesion. A significant increase in adhesion forces was observed as the deposition plate temperature was set slightly above the glass transition temperature of the printing material. Costa et al. [11] modeled the heat transfer mechanisms occurring during deposition: convection with the environment, conduction between adjacent layers and conduction with the deposition plate. The effect on temperature evolution has been discussed and correlated with the interlayer adhesion. Lee et al. [12] analyzed the influence of the environment temperature and heat flux generated by cooling fans. It was found that higher cooling speeds generated better geometric accuracy but lower mechanical strength. McIlroy and Olmsted [13] correlated temperature evolution to the entanglements recovery, finding that more entangled welds could be formed by increasing the plate deposition temperature.

To further understand how the thermal history of the deposited layers is correlated to morphology and mechanical properties of the produced FDM object, the role played by crystallization phenomena, and a molecular weight between the entanglements must be taken into account. To this purpose, in the presented work, the morphology and the mechanical properties of FDM specimens have been correlated to the thermal history experienced by the polymer during deposition, including the role played by the crystallization phenomena and the molecular diffusion in terms of molecular weight between entanglements. A mathematical model of the heat transfer occurring during FDM deposition, including the evolution of the crystallinity degree, has also been proposed and correlated with the experimental observations.

## 2. Materials and Methods

PLA 02-B-0015 filaments (Robotics 3D, Rome, Italy) with a mean diameter of 1.75 mm were used as printing materials. This material showed 0.3 ± 0.05 w% of filler amounts, as detected from thermogravimetric analyses, performed with TG 209 F1 (Netzsch Geretebau, Selb, Germany). A 0.140 mm thickness Kapton^®^ film was used as printing bed material.

The FDM process was conducted with a Makerbot 3D printer (Replicator 2X, Robotics 3D, Rome, Italy), having a 0.4 mm nozzle. All the specimens were produced with 40 mm/s printing speed, without raft and supports. The layer thickness of the first layer was set constant to 0.3 mm; the infill was 99%. The razor direction of the adjacent layer was 90°. Nozzle and printing bed temperatures adopted in this work (T_n_ and T_b_, respectively) are summarized in Table 1.

**Figure 1:**
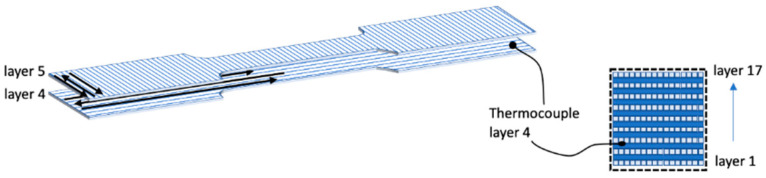
Sketch of the thermocouple positioning during the tests.

T-type thermocouples (d = 120 μm, Omega Engineering, Manchester, UK) were added in parallel with the deposition layer during the deposition to record temperature during the process. Thermocouple datalogger (TC-08, Pico Technology, St Neots, Cambridgeshire, UK) was adopted for temperature recording. Figure 1 shows a schematic representation of the thermocouple positioning between deposited layers.

The specimen geometry was selected according to ANSI D638; in particular, Type V was selected.

Tensile tests were performed on a universal mechanical testing machine (ATSFAAR TC1000, Atsfaar Industries SRL, Segrate, Italy) equipped with a load cell of 1 kN. At least five replicates for each kind of printing material were tested at a constant ramp speed of 0.16 mm/min, and the tensile data were obtained as the average of these five specimens.

The dynamic mechanical analysis (DMA) was performed with PerkinElmer DMA 8000 (Waltham, MA, USA) using the dual cantilever bending method. The temperature scans were performed from room temperature to 120 °C, with 2 °C/min scan rate, a deformation of 50 μm and a frequency of 1 Hz.

Calorimetric analyses were performed by differential scanning calorimeter DSC 822TM (Mettler Toledo Inc., Columbus, OH, USA). The temperature calibration was conducted with the onset extrapolated temperature of the melting transition of indium (156.6 °C, calibration standard, purity >99.999%). 50 mL/min was adopted as a nitrogen flow rate to prevent thermo-oxidative degradation. The melting behavior of the materials was investigated in the range 25–200 °C, with different heating/cooling rates. The latent heat of melting of PLA was 93 J/g [14,15].

A section of the produced specimens was cut along the transversal direction with respect to the deposition direction. The specimens were polished, and the plasticized material was removed following the etching procedure reported elsewhere [16]. The specimens were observed by an optical microscope (BX51, Olympus, Tokyo, Japan) equipped with a digital camera.

Atomic force microscopy (AFM) was adopted for the simultaneous acquisition of morphology and elastic modulus maps. For this purpose, a NanoScope MultiMode V scanning probe microscope (Veeco, Santa Barbara, CA, USA) equipped with HarmoniX tool was adopted. HMX probe cantilevers (10 nm nominal radii, Bruker, Billerica, MA, USA), 44 kHz vertical frequency, and 1140 kHz torsional frequency were selected for the abovementioned analyses. The information about the calibration of the HarmoniX tool and the model adopted for reconstructing the elastic modulus map from the acquired force are reported elsewhere [17,18].

## 3. Model for the Description of Temperature Evolution at the Interface and Crystallization Kinetic

The heat transfer coupled with the latent heat of crystallization governs the deposition:

(1) $$\rho \;Cp\;\frac{\partial T}{\partial t}=k\nabla^{2}T+\lambda \;\rho \frac{\partial x_{c}}{\partial t}\;$$

where *ρ* is the material density (1200 kg m^−3^), *Cp* is the specific heat (1800 J kg^−1^ K^−1^), *k* is the thermal conductivity (0.13 W m^−1^ K^−1^), *λ* is the latent heat of crystallization (93,000 J kg^−1^), *x_c_* is the crystallization degree. In the simulations, the density, the specific heat, the thermal conductivity, and the latent heat of crystallization were considered constant.

**Figure 2:**
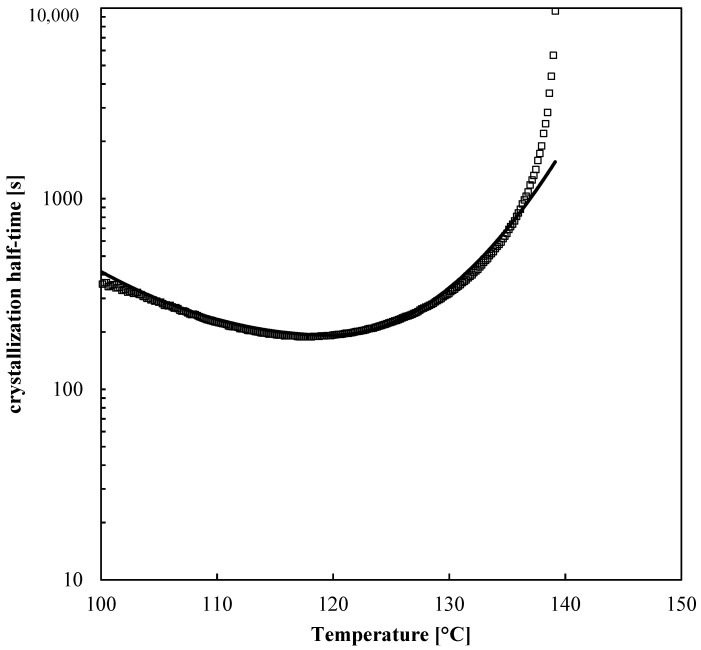
Half crystallization time as a function of temperature (symbols). The crystallization half-time evaluated by the Nakamura approach is also reported (line).

The solidification of the net material was conducted in a DSC apparatus, with liquid nitrogen as a cooling fluid. The material was kept at 200 °C for 10 min to erase previous thermomechanical history and then cooled down with different cooling rates, 20, 40 and 80 °C/min. This procedure allowed determining the crystallization half-time, as shown in Figure 2.

In the temperature ranges of the FDM process, the adopted PLA showed long crystallization times (the minimum crystallization time in about 200 s).

The Nakamura approach for nonisothermal crystallization kinetics was adapted for describing the kinetics of the material adopted in this work [19]. The crystallinity evolution is given in Equation (2):

(2) $$x_{c}=x_{e}\left[1-exp\left(-ln\;2\;{\left(\int_{0}^{t}K\left(T\right)dt\right)}^{n}\right)\right]$$

*x_e_* is the equilibrium crystallinity value; *n* is the Avrami index; when the temperature is constant, *K*(*T*), the kinetic constant is equal to the reciprocal of crystallization half-time. *K*(*T*) shows a maximum in *T_max_* between the melting temperature (*T_m_*) and the glass transition temperature. Generally, *K*(*T*) is a bell-shaped curve; however, a symmetric curve does not allow a good description of the experimental data. For this reason, the function *K*(*T*) is allowed to be nonsymmetric by adopting the expression given in Equation (3), where *D*, the full width at half maximum, can assume the value of *D_m_* for *T* < *T_max_* and *D_h_* for *T_m_* < *T* < *T_max_*.

(3) $$K\left(T\right)=\begin{array}{c} K_{0}exp\left(\frac{-4ln\;2\;{\left(T-T_{max}\right)}^{2}}{D_{h}^{2}}\right)\;if\;T_{m}<T<T_{max}\\ K_{0}exp\left(\frac{-4ln\;2\;{\left(T-T_{max}\right)}^{2}}{D_{m}^{2}}\right)\;\;\;if\;T<T_{max}\; \end{array}$$

*K*(*T*) will become symmetric if *D_h_* = *D_m_*. *K*_0_ is the maximum value of *K*(*T*). The values of the adopted parameters are summarized in Table 2 The comparison among experimental data and the crystallization half-time evaluated as reciprocal of *K*(*T*) is shown in Figure 2.

**Figure 3:**
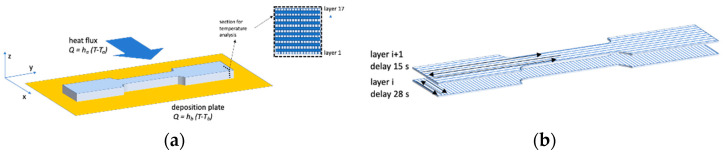
(**a**) Sketch of the boundary conditions adopted in the model for the simulation of the temperature evolutions during deposition. (**b**) Sketch of the deposition direction and delay times adopted for each layer.

The boundary conditions are sketched in Figure 3a.

Two heat transfer coefficients were adopted, accounting for the heat exchange between deposited layers and the deposition plate and the heat exchange between deposited layers and the surrounding environment. Figure 3a also shows the section where the temperature evolution was analyzed (dash-dot line).

The software adopted for simulation was developed on purpose and written in LabView. The solution of the differential equations is based on a finite difference method.

The domain is 2D with a grid spacing of Δs = 10 μm in both directions (340 × 340 points in our geometry). The time step was set at Δ*t* = 5 × 10^−4^ s. The energy balance equation is solved at each time-step only in the space occupied by the polymer. The thermal conditions at the boundaries (either for the layers in contact with the bottom plate and for the layers in contact with the air) are given in Equation (4):

(4) $$-k\frac{\partial T}{\partial m}=h\left(T-T_{e}\right)\;$$

where *m* identifies the outer-pointing normal direction, *h* is a heat transfer coefficient (*h_a_* = 30 W m^−1^ K^−1^ is the heat transfer coefficient with air, and *h_b_* = 600 W m^−1^ K^−1^ is the heat transfer coefficient with the deposition plate, see Figure 3) and *T_e_* is the temperature of the element in contact with the polymer.

**Figure 4:**
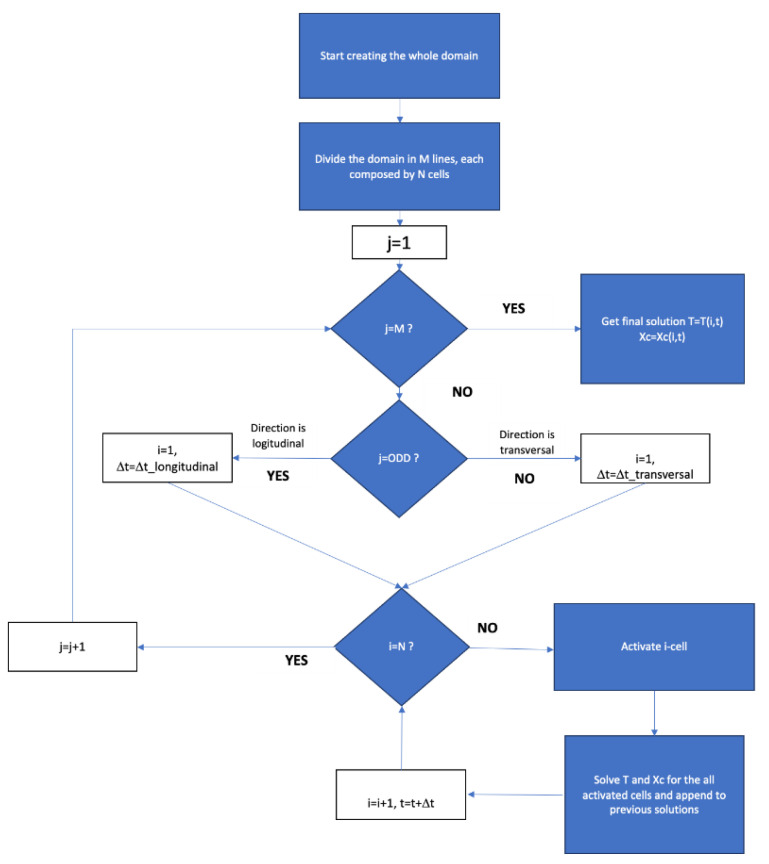
Schematic representation of the process simulation flow chart.

The addition of a new filament was modeled by considering a new hot square (side 200 μm) at an initial temperature of T_i_ = 200 °C, which expands the space occupied by the polymer. The time step needed to add a new hot square was chosen to describe the real process (in our case, Δt_odd_ = 0.085 s for the odd layers and Δt_even_ = 1.5 s for the even ones). At each time step, the software computes the increase of crystallinity on the basis of the values of temperature and crystallinity at the same point calculated at the previous time, and then the new temperature. For describing the two deposition directions, with a raster angle of 90°, two delay times were introduced, 28 s for odd-layer and 15 s for the even one (see Figure 3b). Figure 4 shows the flow chart of the simulation process.

## 4. Results

**Figure 5:**
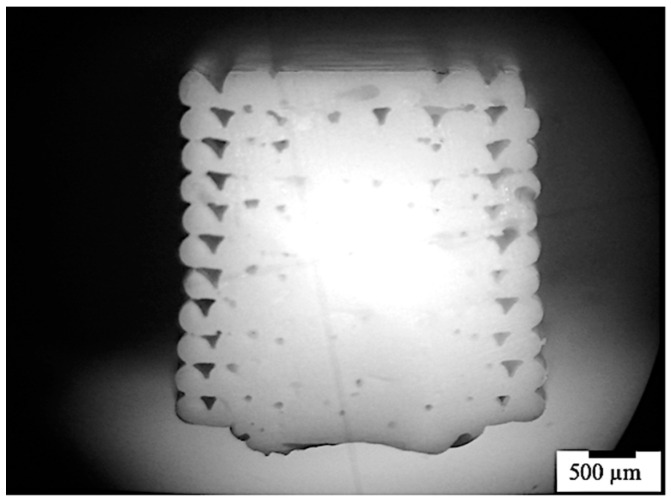
Optical micrograph of the specimen obtained with T_n_ = 230 °C and T_b_ = 90 °C. The bottom part corresponds to the layer which contacts the deposition plate.

The morphology of the specimens produced by FDM was analyzed by optical microscope. Figure 5 shows the optical micrograph of the specimen obtained with T_n_ = 230 °C and T_b_ = 90 °C. The layers closer to the deposition plate showed a dense structure, with a smaller number of pores than the layers far from the deposition plate. The layers closer to the deposition plate experienced a slow cooling rate; thus, the diffusion at the interface between adjacent layers was favored. Vice versa, the layers at a larger distance from the deposition plate were subjected to faster cooling; thus, the molecular diffusion may have been limited.

**Figure 6:**
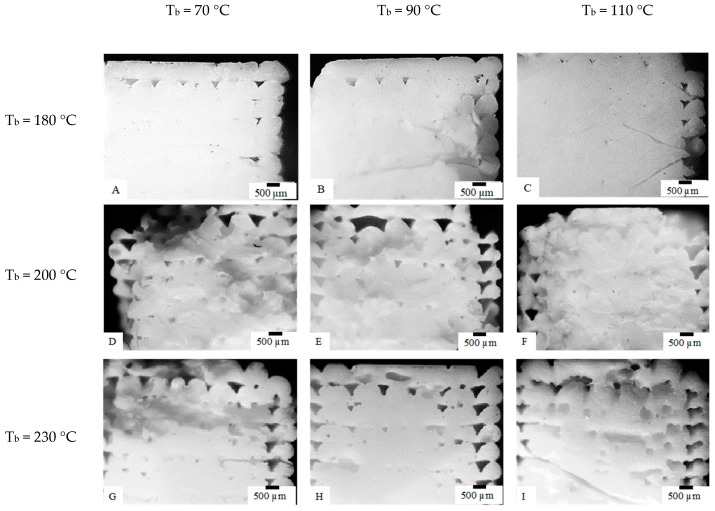
Optical micrographs of the specimens obtained at different T_n_ and T_b_. The part of the specimen farer from the deposition plate has been shown. T_n_ and T_b_ have been also indicated in the figure (see Table 1 for operative conditions).

The temperature field was expected to have a significant influence on the specimen compactness. Figure 6 shows the optical micrographs of the topmost specimens (i.e., the part of the specimen farther from the plate deposition) obtained with different T_n_ and T_b_, as previously summarized in Table 1.

The micrographs related to specimens F and I, obtained with both high T_n_ and T_b_, show a more compact structure with respect to the specimens obtained with lower temperatures, D and G. The increase of T_n_ seemed to be more efficient in increasing the compactness than T_b_. However, the specimens obtained with T_n_ = 180 °C showed a negligible dependence of morphology on T_b_; furthermore, they seemed to be more compact and present less distortion than the specimens obtained with T_n_ = 200 °C and 230 °C.

**Figure 7:**
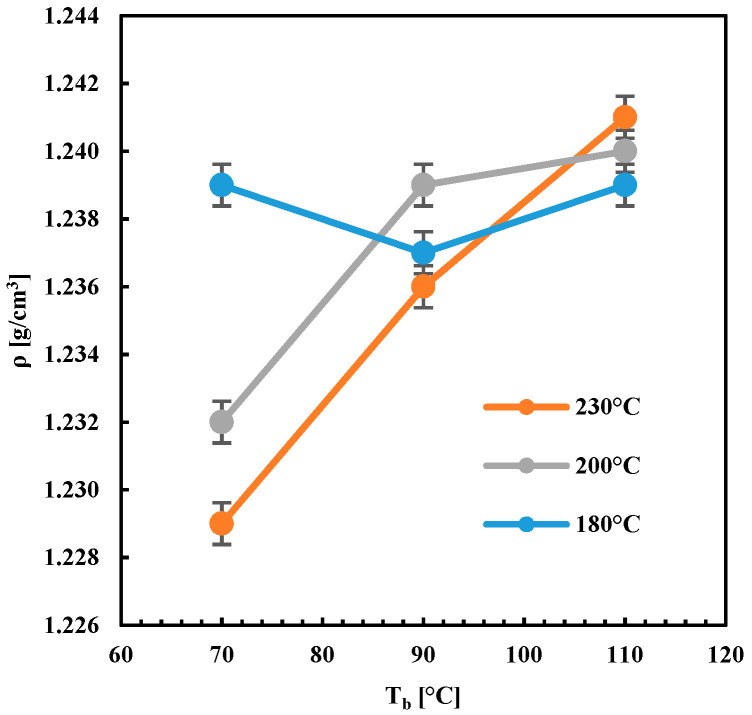
Bulk density of the specimens obtained with different T_n_ and T_b_.

Figure 7 shows the density of the specimens obtained by FDM as a function of both T_n_ and T_b_. The density of the specimens increased with the temperature T_b_ when the higher nozzle temperatures were adopted (T_n_ = 200 °C and 230 °C). When T_n_ = 180 °C, the density of the specimens did not depend on the plate deposition temperature T_b_. This behavior was consistent with the microscopy observations of Figure 6. The high values of density with T_n_ = 180 °C, also at low T_b_, could be ascribed to the mechanical deformation experienced by the filament during deposition. The FDM proceeds with a constant flow rate [20]: under such a condition, the pressure at the nozzle exit may increase on decreasing the nozzle temperature. The higher pressure would induce the deformation of the filament and the reduction of void degree.

**Figure 8:**
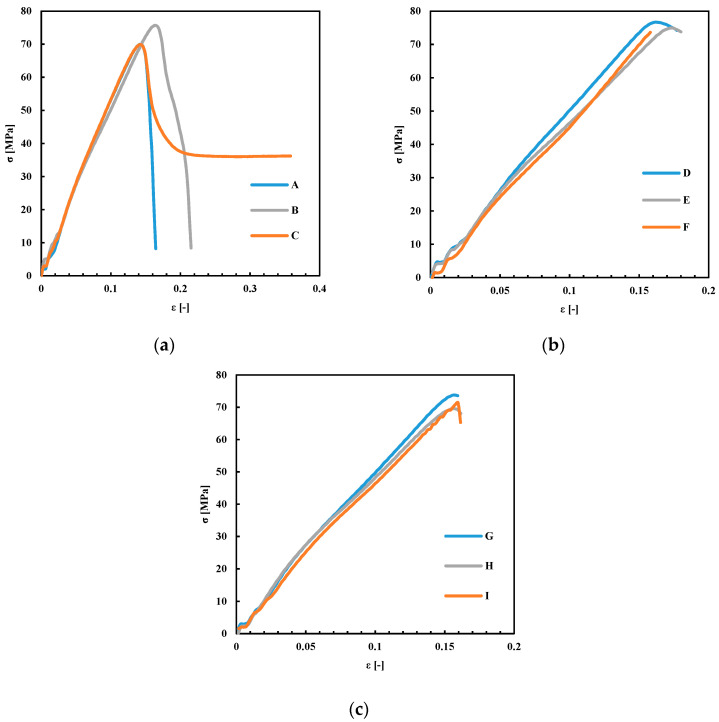
Stress–strain plots for the specimens obtained by FDM with different T_b_. (**a**) T_n_ = 180 °C; (**b**) T_n_ = 200 °C; (**c**) T_n_ = 230 °C. A,D,G: T_b_ = 70 °C; B,E,H: T_b_ = 90 °C; C,F,I: T_b_ = 110 °C.

Figure 8 shows the stress–strain plots obtained for the specimens produced with different nozzle and plate deposition temperatures. Table 3 summarizes the results obtained from the tensile analyses.

The results reported in Figure 8 and summarized in Table 3 show that the tensile strain at the tensile strength, ε, was not sensitive to the changes in T_n_ and T_b_. The total extension achieved at the end of the test, ε_t_, increased with T_b_ at the lowest T_n_, whereas, at higher T_n_, it was not sensitive to T_b_. Interestingly, the specimen C showed the bigger ε_t_ with respect to all the other samples, which generally showed a fragile behavior. This behavior could be due to an enhancement of the interlayer diffusion induced by a higher T_b_. This effect was not observed in the specimens obtained with the same T_b_ (110 °C) and higher T_n_. Probably, higher nozzle temperatures induced a partial degradation of the material that induced fragility in the final part.

**Figure 9:**
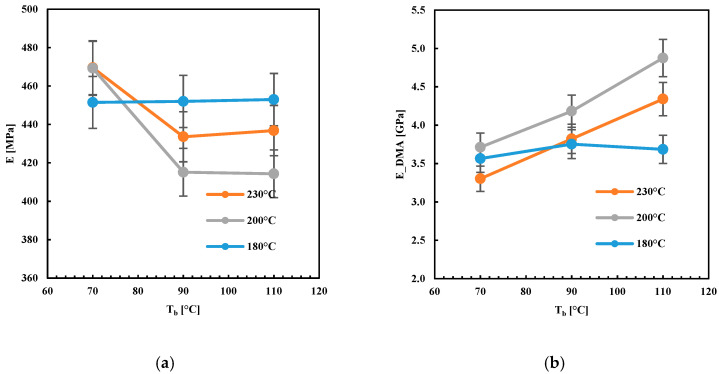
Elastic modulus (**a**) from tensile and (**b**) DMA analyses plot versus the plate deposition temperature T_b_ at different nozzle temperatures.

Figure 9 shows the values of the elastic modulus, E, evaluated by two different kinds of analysis, tensile and DMA, as a function of the temperatures adopted during FDM, T_n_ and T_b_.

The tensile elastic modulus decreased with T_b_ when high T_n_ was adopted. The elastic modulus evaluated by DMA showed a different behavior; it decreased with T_n_ and increased with T_b_. For T_n_ = 180 °C, the moduli, both tensile and that one measured by DMA, did not depend on T_b_. A similar behavior, with respect to the temperatures T_n_ and T_b_, was observed for the density. This suggests that both these two properties (elastic modules evaluated by DMA and density) depended on the transient temperature field established within the deposited layers.

**Figure 10:**
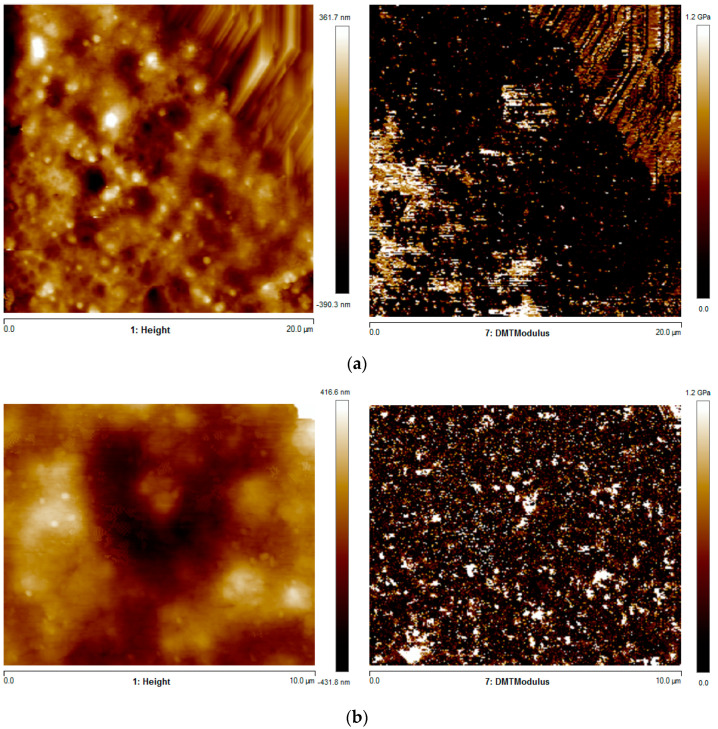
AFM height and elastic modulus maps of the specimens F in the (**a**) topmost and (**b**) central parts.

AFM analyses allowed detecting the adhesion quality at the interface between adjacent layers by the simultaneous acquisition of morphology and elastic modulus maps. Figure 10 shows the AFM heigh (morphology) and elastic modulus (DMT modulus) maps of the specimens obtained with T_n_ = 200 °C and T_b_ = 110 °C (F in Table 1). The analyses refer to the topmost (the part at the larger distance form the deposition plate) and central parts of the specimen where interfaces were detected.

The AFM map revealed a morphology typical of amorphous samples, with globular structures generally developed when the sample experiences high cooling rates [21]. The presence of poor adhesion between adjacent layers could be detected in the topmost part of the specimen; in particular, the elastic modulus map revealed the presence of a region with modulus lower than those of the adjacent areas. Such a region could be attributed to poor adhesion, which led to the low mechanical property. In the central part of the specimen, the homogeneous distribution of the elastic modulus was due to a more efficient interlayer diffusion. The average elastic modulus was evaluated in each position: 220 MPa in the topmost part and 500 MPa in the central part.

**Figure 11:**
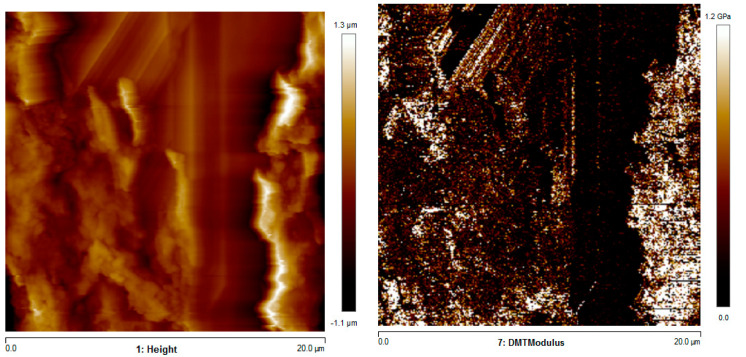
AFM height and elastic modulus maps of the specimens C.

Figure 11 shows the AFM heigh (morphology) and elastic modulus (DMT modulus) maps of the specimens obtained with T_n_ = 180 °C and T_b_ = 110 °C (C in Table 1).

The elastic modulus was lower at the interface between adjacent layers than the other regions. The average elastic modulus was 520 MPa. Generally, the values of elastic modulus provided by AFM agreed with the ones measured by tensile tests. Additionally, the increase of the nozzle temperature induced a decrease in the elastic modulus.

Morphological analyses do not allow to capture the phenomena influencing the mechanical performances; the orientation and the crystallinity degree achieved during the process must be considered too. In turn, crystallinity and residual orientation depend on the temperature field undergone by the molecules during deposition. For this reason, temperature evolutions during deposition were recorded and correlated with the crystallization process and the molecular weight between entanglement (*M_e_*), which gives information about the residual orientation.

**Figure 12:**
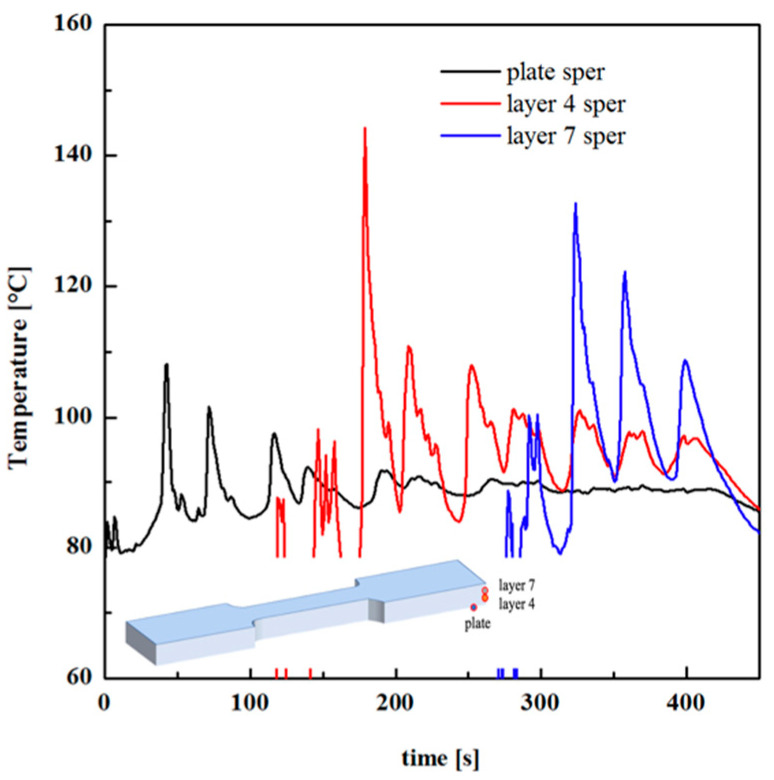
Temperature evolutions recorded at different layers. A sketch of the layers is also shown.

Figure 12 shows temperature recordings during FDM production of specimen E, with T_n_ = 200 °C and T_b_ = 90 °C. The temperature was recorded in three positions, namely the deposition plate, layer 4, and layer 7 (the part is composed of 17 layers).

Temperature–time evolutions allow determining the cooling rate during deposition, that, for the case shown in Figure 12 (T_b_ = 90 °C), is 35 °C/s, at the first contact of the polymer with the previously deposited layer. The cooling rate was 80 °C/s for the case D, and 20 °C/s for case F. Obviously, the cooling rate decreased as the difference between the nozzle temperature and the plate deposition temperature decreased. Thus, the tests conducted with higher nozzle temperature (i.e., T_n_ = 230 °C) showed higher cooling rates with respect to the cased with T_n_ = 200 °C. Temperature recordings reveal the partial reheating of the previously deposited layer during the deposition of the subsequent layer. However, the reheating was also due to the deposition of the filament on the neighbor areas. Considering temperature recording related to layer 4, in the time range 180–200 s, after the first contact of the melt with the thermocouple position, the temperature profile showed three shoulders due to the deposition of the filament on the neighbor areas. The new temperature peak at 210 s was due to the deposition of the subsequent layer (layer 5).

**Figure 13:**
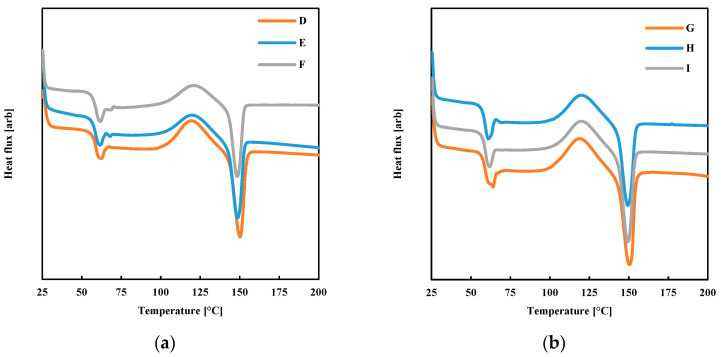
Calorimetric analyses were performed on the specimens obtained by FDM with different T_b_. (**a**) T_n_ = 200 °C; (**b**) T_n_ = 230 °C.

Figure 13 shows the calorimetric analyses conducted on the specimens produced by FDM to measure the average crystallinity degree.

All the specimens showed a crystallinity degree below 3%. Thus, the mechanical performances of FDM produced parts must be determined by the number of entanglements per chain formed upon interfacial diffusion [22], in other words, by the molecular weight between entanglements (*M_e_*).

The mutual uncrossability of polymer chains generates topological constraints, generally called entanglements, which effectively restrict individual chain conformations in a curvilinear tube-like region surrounding each chain. Large-scale motion was promoted via reptation [23], an effective one-dimensional diffusion of a chain along its tube axis. In reptation theory, each polymer is supposed to move in a tube around a Gaussian path in space. A rubbery network-like structure forms among the polymer chains, the so-called “entanglement network”. Such a rubbery network reflects the complex molecular topology and affects polymer dynamic and rheology.

The key to ensuring the strength of the final printed part is successful diffusion and re-entanglement of the polymer melt across the interfaces between adjacent layers [24]. The value of *M_e_* can be influenced by the stretch the molecules experience during a process. The shear experienced by the melt during the extrusion process may stretch the polymer molecules; as a consequence, the entanglement density reduces, and *M_e_* increases [25].

*M_e_* is given by Equation (5), where *G_e_* is modulus in the rubbery plateau region obtained from the oscillatory analysis [26]:

(5) $$M_{e}=\frac{3N_{L}k\rho T_{g}}{G_{e}}$$

*ρ* is the polymer melt density, *N_L_* is the Avogadro number, *k* is the Boltzmann constant, *T_g_* is the temperature at the glass transition determined from the DSC analyses.

**Figure 14:**
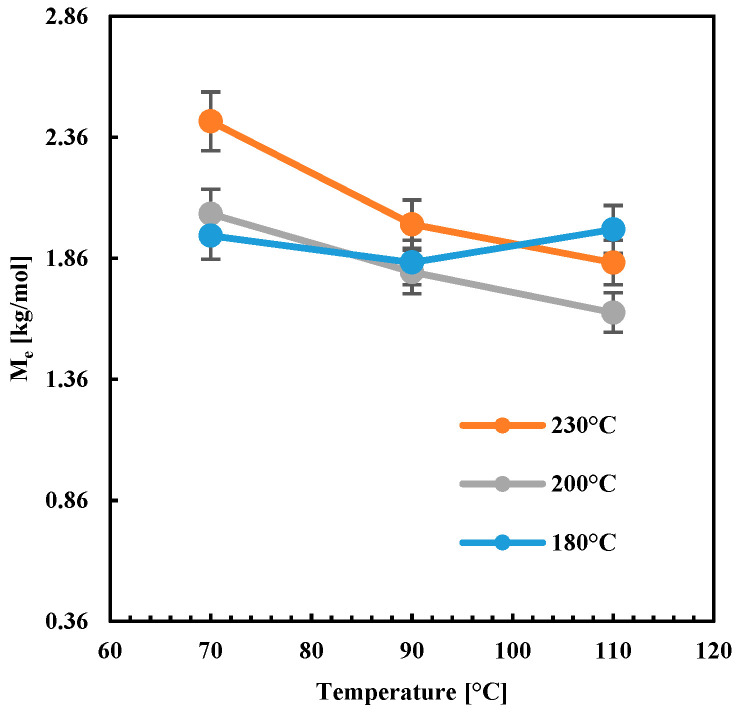
Molecular weight between entanglement plot versus the plate deposition temperature T_b_ at different nozzle temperatures.

Figure 14 shows *M_e_* in the function of T_b_ and T_n_. *M_e_* decreased with T_b_ increase when high nozzle temperatures were adopted (T_n_ = 200 °C and 230 °C), whereas, for low nozzle temperature, *M_e_* did not depend on T_b_. The increase of T_n_ from 200 to 230 °C induced an increase of *M_e_* at all the temperatures T_b_.

As mentioned above, the *M_e_* increase was due to an increase in the residual stretch; this means that specimens A, D and G show higher residual stretch than the other specimens. The levels of residual stretch were an indication of the interlayer adhesion quality: higher were the levels of residual stretch, lower was the interlayer molecular diffusion, and poorer was the interlayer adhesion quality. When high nozzle temperatures were adopted, the flow was more efficient in orienting molecules. However, the increase of T_b_ allows for molecular relaxation since molecules experience high-temperature ranges for longer times. Thus, the reduction of *M_e_* with the increase of T_b_ was consistent with the expectations. The parts showing lower *M_e_* were characterized by better interlayer adhesion than the part characterized by higher *M_e_* [15]. This observation was also consistent with those reported in the literature about the force necessary for the debonding of adjacent layers: at constant plate deposition temperature, the higher was the nozzle temperature, the higher was the force required for debonding [9]. This confirmed that the method based on the evaluation of the molecular weight between entanglements for the evaluation of the adhesion quality was reliable.

## 5. Discussion

In this section, the results in terms of the mechanical performance, crystallinity, and molecular weight between entanglements are discussed on the basis of the simulation outcomes concerning temperature field and crystallinity prediction.

**Figure 15:**
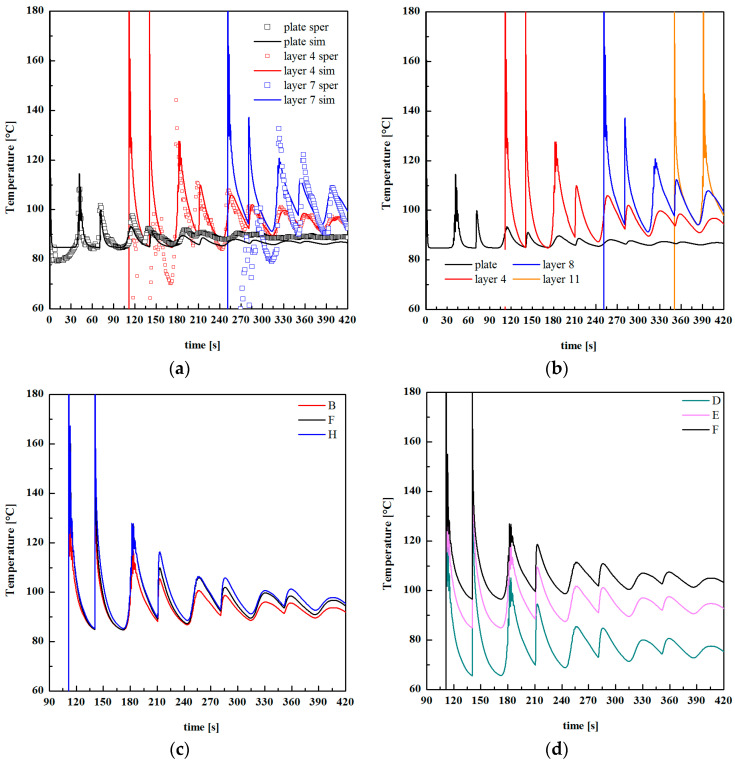
(**a**) Comparison among temperature evolution recorded during FDM case F (symbols) and the simulated temperature evolution (lines); (**b**) temperature evolutions evaluated by simulation of different layers for the case F; (**c**) temperature evolutions evaluated by simulation of different layers for the cases B, F and G; (**d**) temperature evolutions evaluated by simulation of different layers for the case D and F.

Figure 15a shows the comparison among temperature evolution recorded during FDM test F (T_n_ = 200 °C; T_b_ = 90 °C) and the simulated temperature evolution. Three simulated temperature evolutions of different layers are shown: the deposition plate, layer 4 and layer 7.

The simulated time–temperature evolution was consistent with the experimental ones, particularly with the reheating effect recorded when a new layer was deposited on the previous one. In addition, temperature peaks increased with the distance from the deposition plate since the previously deposited layers act as thermal insulant for the new layer, slowing down the cooling rate. Temperature evolutions of different layers for the case F (see Figure 15b) confirm this finding. Figure 15c,d shows temperatures evolutions for layer 4 of the cases with constant T_b_ = 90 °C and different nozzle temperatures, and with constant T_n_ = 200 °C and different plate deposition temperatures, respectively. At a fixed T_n_, the higher was T_b_; the longer was the molecule residence time within the temperature range of 100–140 °C. A further T_n_ increase induces only a slight variation in the time the molecules spend at high temperatures. Furthermore, the higher was T_b;_ the longer was the time the molecules spend within the temperature range (100–140 °C), which guaranteed high molecular mobility. This finding explains the behavior of density and *M_e_* observed in Figure 7 and Figure 14. The longer was the time the molecules spend within the temperature range of 100–140 °C; the higher was the molecular mobility, the more efficient was the interlayer diffusion. This induced the formation of a more compact structure, with higher density and with low residual stretch, i.e., low *M_e_*. It must be noticed that density was related to the crystalline degree: the increase of crystalline degree induced a density increase. The behavior of density with T_b_ was also consistent with an increase of crystallinity induced by a longer residence time of the molecules within the crystallization temperature range on increasing T_b_.

**Figure 16:**
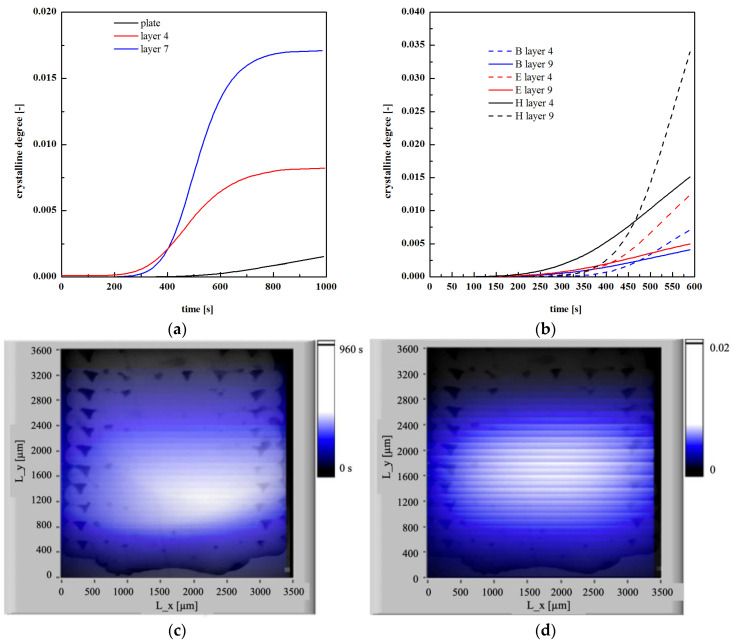
(**a**) Crystallinity evolutions of different layers for the case E evaluated by simulation; (**b**) crystallinity degree evaluated at layer 4 and layer 9 for the cases B, E and H; (**c**) time the molecules spend above 90 °C for the case E; (**d**) crystallinity measured after 1000 s for the case E. The optical micrograph of the specimen E is also embedded in the graphs.

Figure 16a shows the crystallinity evolution of different layers for the case E. The video S1 in the supplementary material gives the evolutions of temperature, crystallinity and time spend above 90 °C for the case E.

The layer close to the deposition plate showed negligible crystallinity degree, reaching less than 0.2% within 1000 s. Layer 7 reached a 1.6% crystalline degree within 1000 s. The increase of the crystallinity toward the specimen center is also represented in Figure 16c; Figure 16d shows the overall residence time the molecules spend above 90 °C. The distribution of time the molecules spent above a certain temperature was quite similar to the optical micrograph, also shown in the figure: this confirms that the compactness depends on the time the molecules spent above a certain temperature, during which interlayer diffusion was allowed. In the specimen center, the crystallization was higher due to a longer residence time within the crystallization temperature range. However, the overall crystallinity was very low, with an average value smaller than 1%. Figure 16b shows the crystallinity evolution obtained at layer 4 and layer 9 for B, E and H cases. The crystallinity degree was very low for all the analyzed cases; it never exceeded 3.5%, consistently with the experimental observations. The simulations revealed that the crystallinity increased with the nozzle temperature due to a longer residence time of the layers within the crystallization temperature range. For the same reason, crystallinity became higher toward the specimen center (see crystallinity evolutions for layer 9).

The layer close to the deposition plate showed negligible crystallinity degree, reaching less than 0.2% within 1000 s. Layer 7 reached a 1.6% crystalline degree within 1000 s. The increase of the crystallinity toward the specimen center is also represented in Figure 16c; Figure 16d shows the overall residence time the molecules spend above 90 °C. The distribution of time the molecules spent above a certain temperature was quite similar to the optical micrograph, also shown in the figure: this confirms that the compactness depends on the time the molecules spent above a certain temperature, during which interlayer diffusion was allowed. In the specimen center, the crystallization was higher due to a longer residence time within the crystallization temperature range. However, the overall crystallinity was very low, with an average value smaller than 1%. Figure 16b shows the crystallinity evolution obtained at layer 4 and layer 9 for B, E and H cases. The crystallinity degree was very low for all the analyzed cases; it never exceeds 3.5%, consistent with the experimental observations. The simulations reveal that the crystallinity increased with the nozzle temperature due to a longer residence time of the layers within the crystallization temperature range. For the same reason, crystallinity became higher toward the specimen center (see crystallinity evolutions for layer 9).

## 6. Conclusions

Fused polymeric filament deposition is a bottom-up based technique allowing the construction of objects layer by layer. This approach, although highly flexible and able to produce objects with very complex shapes, does not lead to obtaining parts showing high mechanical performances. The formation of the object via successive depositions of fused filament on layers undergoing solidification is the main cause of the poor mechanical resistance and the short lifetime of the part. This work was intended to get insight and relate the phenomena occurring during fused PLA deposition and find criteria to enhance mechanical performances of a standard specimen (Type V, ANSI D368). To this purpose, an experimental methodology able to determine the temperature field undergoing by molecular chains during the process and to relate it to the object morphology and to the interlayer diffusion degree was setup. Fused PLA deposition was conducted on a commercial 3D printer, with nozzle temperature ranging from 180 to 230 °C, and temperature of the deposition plate ranging from 70 to 110 °C. Optical microscopy, elastic modulus analysis, and density measurement allowed to emphasize the role played by the temperature field developed within the sample on the morphology and the mechanical characteristics of the specimen.

The AFM map revealed a morphology typical of amorphous samples, with globular structures generally developed in positions where the sample experienced high cooling rates. The presence of poor adhesion between adjacent layers was detected in the topmost part of the specimen, where the elastic modulus was 220 MPa. The central specimen part showed a homogeneous distribution of the elastic modulus, with an average value higher than those measured in the topmost part of the specimen (500 MPa).

The calorimetric analysis revealed that the crystallinity degree of all the specimens was below 3%. The mechanical performances of the produced specimens were then related to the molecular weight between entanglements, measuring the number of entanglements per chain formed upon interfacial diffusion. A successful diffusion and re-entanglement of the polymer melt across the interfaces between adjacent layers was the key to ensure the strength of the final printed part.

The solution of a mathematical model describing the transient heat transfer during the fused PLA deposition and accounting also for solidification and the nonisothermal crystallization kinetics according to the Nakamura approach confirmed the sawtooth temperature profile experimentally detected during the filament deposition, and the results of simulations were related to the mechanical performances, the morphology, and the molecular weight between entanglements of the parts obtained by fused filament deposition. The parts of the specimen which experienced high temperatures (between the glass transition and the crystallization temperature) for a time range sufficiently long showed high compactness and a low molecular weight between entanglements, thus, a sufficient interlayer diffusion degree. This is consistent with the higher modulus determined by DMA. The central parts of the specimens are also characterized by higher levels of crystalline degree, as determined by simulation, even if the overall crystallinity degree is small for all the analyzed conditions.

## Figures and Tables

**Table 1:** Nozzle and printing bed temperatures (T_n_ and T_b_, respectively) were adopted during fused filament deposition (FDM) of polylactic acid (PLA).

Sample N.	T_n_ (°C)	T_b_ (°C)
A	180	70
B		90
C		110
D	200	70
E		90
F		110
G	230	70
H		90
I		110

**Table 2:** Parameters adopted for the description of the crystallization kinetic by the Nakamura approach.

*Ko*	5.0 × 10^−3^	1/s
*x_c_*	0.35	-
*T_max_*	122	°C
*D_m_*	33	°C
*D_h_*	23	°C
*n*	3	-

**Table 3:** Experimental results of tensile tests performed on specimens listed in Table 1 (σ_M_ is the tensile strength, ε is the tensile strain at the tensile strength, ε_t_ is the total extension achieved at the test end).

Sample N.	E (MPa)	σ_M_ (MPa)	ε (−)	ε_t_ (−)
A	451 ± 10	69.5 ± 0.7	0.14	0.16
B	452 ± 10	75.7 ± 0.7	0.17	0.21
C	453 ± 10	69.0 ± 0.5	0.14	0.36
D	469 ± 15	76.5 ± 0.7	0.16	0.18
E	415 ± 10	74.6 ± 0.7	0.17	0.17
F	414 ± 10	73.6 ± 0.7	0.16	0.18
G	469 ± 10	73.7 ± 0.6	0.15	0.16
H	433 ± 10	70.0 ± 0.5	0.16	0.16
I	436 ± 10	71.4 ± 0.5	0.16	0.16

## Data Availability

Not applicable.

## Supplementary Materials

The following are available online at https://www.mdpi.com/2073-4360/13/3/399/s1, Video S1: Simulated temperature, crystallinity and time spend above 90 °C for the case E.
